# ‘Physio-EndEA’ Study: A Randomized, Parallel-Group Controlled Trial to Evaluate the Effect of a Supervised and Adapted Therapeutic Exercise Program to Improve Quality of Life in Symptomatic Women Diagnosed with Endometriosis

**DOI:** 10.3390/ijerph19031738

**Published:** 2022-02-02

**Authors:** María del Mar Salinas-Asensio, Olga Ocón-Hernández, Antonio Mundo-López, Carolina Fernández-Lao, Francisco M. Peinado, Carmen Padilla-Vinuesa, Francisco Álvarez-Salvago, Paula Postigo-Martín, Mario Lozano-Lozano, Ana Lara-Ramos, Manuel Arroyo-Morales, Irene Cantarero-Villanueva, Francisco Artacho-Cordón

**Affiliations:** 1 Department of Physiotherapy, University of Granada, E-18016 Granada, Spain; carolinafl@ugr.es (C.F.-L.); paulapostigo@ugr.es (P.P.-M.); mlozano@ugr.es (M.L.-L.); marroyo@ugr.es (M.A.-M.); irenecantarero@ugr.es (I.C.-V.); 2 Biohealth Research Institute in Granada (ibs.GRANADA), E-18012 Granada, Spain; ooconh@ugr.es (O.O.-H.); franciscopeinado@correo.ugr.es (F.M.P.); carmenpadvin@ugr.es (C.P.-V.); 3 “Cuídate” Support Unit for Oncology Patients (UAPO), Sport and Health University Research Institute (iMUDS), E-18016 Granada, Spain; 4 Gynaecology and Obstetrics Unit, ‘San Cecilio’ University Hospital, E-18016 Granada, Spain; 5 Clinic Psychology Center Alarcón (CPCA), E-18004 Granada, Spain; antonio@alarconpsicologos.com; 6 Department of Physiotherapy, European University of Valencia, E-46010 Valencia, Spain; francisco.alvarez2@universidadeuropea.es; 7 Gynaecology and Obstetrics Unit, ‘Virgen de las Nieves’ University Hospital, E-18012 Granada, Spain; ana_lara_ramos@hotmail.com; 8 Department of Radiology and Physical Medicine, University of Granada, E-18016 Granada, Spain; 9 CIBER Epidemiology and Public Health (CIBERESP), E-28029 Madrid, Spain

**Keywords:** endometriosis, physiotherapy, therapeutic exercise, quality of life, motor control, pain

## Abstract

Aim: The ‘Physio-EndEA’ study aims to explore the potential benefits of a therapeutic exercise program (focused on lumbopelvic stabilization and tolerance to exertion) on the health-related quality of life (HRQoL) of symptomatic endometriosis women. Design: The present study will use a parallel-group randomized controlled trial design. Methods: A total of 22 symptomatic endometriosis women will be randomized 1:1 to the Physio-EndEA or usual care groups. The ‘Physio-EndEA’ program will consist of a one-week lumbopelvic stabilization learning phase followed by an eight-week phase of stretching, aerobic and resistance exercises focused on the lumbopelvic area that will be sequentially instructed and supervised by a trained physiotherapist (with volume and intensity progression) and adapted daily to the potential of each participant. The primary outcome measure is HRQoL. The secondary outcome measures included clinician-reported outcomes (pressure pain thresholds, muscle thickness and strength, flexibility, body balance and cardiorespiratory fitness) and patient-reported outcomes (pain intensity, physical fitness, chronic fatigue, sexual function, gastrointestinal function and sleep quality). Discussion: Findings of this study will help to identify cost-effective non-pharmacological options (such as this exercise-based intervention) that may contribute to the improvement of HRQoL in symptomatic endometriosis women.

## 1. Introduction

Endometriosis is considered among the most prevalent disease conditions in women of childbearing age. Although the numbers are still unknown, it has been estimated that 8–11% of women of reproductive age have endometriosis (with or without a clinical diagnosis) [1], counting for approximately 176 million affected women worldwide [2], with a peak incidence in women who are in their thirties and forties [3]. Moreover, this percentage increases, reaching 30–40%, when considering those women with a history of infertility [1]. It is an oestrogen-dependent female disease characterized by the proliferation of endometrial glands and stroma outside the uterine cavity [4]. In general, depending on the site of implementation, lesions can be differentiated into ovarian (ectopic tissue adhered to the ovaries), peritoneal or deeply infiltrating lesions [5].

Although endometriosis was described for the first time by Carl Von Rokitansky more than 150 years ago [6], little is known about the aetiology and pathogenesis of the disease and, therefore, about therapeutic options. Despite that some theories have tried to explain the development of these ectopic lesions [7], it is currently gaining recognition the endocrine-disrupting hypothesis that suggests that human exposure to synthetic chemicals with endocrine mimicking properties may be underlying the pathogenesis of endometriosis [8]. In this regard, it has been recently suggested that foetal exposure to diethylstilbesterol, a known endocrine-disrupting chemical, may underlie the development of endometriosis in adulthood [9]. In any case, an in situ chronic oxidative/inflammatory milieu originated by these ectopic lesions is frequently observed, which may lead to an altered microenvironment that includes, among others, the activation of epithelial-mesenchymal transition cell-signalling pathways [7]. Moreover, independently of their implementation site, endometriotic lesions are frequently detected to be vascularized and innervated [10,11]. The development of these nerve fibres (responsible for conveying nociceptive signals), but not the extent, location or type of endometriosis found at laparoscopy seems to be related to the severity of pain [12]. Hence, the affected women usually report dysmenorrhea (i.e., painful menstruation), dyspareunia (i.e., painful sexual intercourse), dyschezia (i.e., painful defecation), dysuria (i.e., painful urination) and, in general, chronic pelvic pain (i.e., pain perceived to originate in the pelvis lasting for longer than 6 months) [13].

In fact, pain, considered in its multiple versions, is acknowledged to be the most common and more disabling symptom of endometriosis [14,15], although the burden of endometriosis symptoms is highly variable between patients [16]. Pain is suggested to underlie the reduced level of physical activity [17,18], and the high prevalence of reported chronic fatigue [18,19,20], as well as the broad burden of disabilities described for some of these affected women [21]. In this regard, it has been published a relevant worse impact on everyday activities [21,22,23], sleep quality [14,17,20], relationship with their partner and reproductive planning [24,25], emotional and mental health [26,27], education [28,29], work productivity [30,31,32,33] or social life [28,29] that, in combination, leads to a significant reduction in health-related quality of life (HRQoL) [14,18,20,24,31,34]. Even more, endometriosis is acknowledged to be a risk factor for gynaecological cancer [4]. As a result, a systematic review confirms a substantial economic burden associated with endometriosis [35]. Although they greatly varied by country, direct costs (including inpatient, outpatient, surgery, drug and other healthcare service cost) ranged from USD 1109 per patient per year in Canada to USD 12,118 in the USA. Indirect costs (related to the loss of productivity at work) ranged from USD 3314 per patient per year in Austria to USD 15,737 in the USA [35].

In spite of the medical management of endometriosis having not been fully standardized worldwide, treatment schedules often include a palliative pharmacological control of pain symptoms (analgesics and oral contraceptives being the most commonly indicated) and surgery for resection of lesions, although with an elevated risk for recurrence [4]. Nevertheless, this therapeutic approach is clearly insufficient to manage the burden of symptoms in many women with endometriosis [4,36]. In this context, rehabilitation, through the vast array of therapeutic strategies for pain control might offer a substantial benefit to ameliorate pain-related disabilities and to improve HRQoL in endometriosis women. Indeed, a case–control study focused on visceral osteopathy [37], a retrospective study evaluating electrical muscle stimulation [38] and two randomized controlled trials exploring the potential benefits of pulsed high-intensity laser therapy [39] and yoga [40] have reported a significant improvement in HRQoL in these patients, although the contribution to individual occupational roles have not been addressed yet. Moreover, in contrast to osteopathy (with limited scientific evidence) or laser therapy (with non-standardized dosimetry), therapeutic exercise has been widely demonstrated to induce either soft tissue remodelling [41] and modifications in the nervous system [42], of particular interest in endometriosis given the elevated prevalence of central sensitization in these patients [43,44]. Additionally, it has been shown that regular physical exercise also exerts a protective effect against diseases that involve oxidative and/or inflammatory processes since it induces an increase in the systemic levels of mediators with anti-oxidant and anti-inflammatory properties [45]. In fact, a recent study has shown that the practice of regular physical exercise increases anti-oxidative responses in experimentally induced endometriosis in rats [46]. However, the limited number of publications lead the European Society of Human Reproduction and Embryology (ESHRE) to declare that the usefulness of physical practice for primary prevention of endometriosis is uncertain [47].

Chronic pelvic and visceral pain are typically referred to as somatic structures (skin, subcutis and muscle) leading to significant trophic changes of soft tissues, such as decreased muscle thickness [48], probably due to pain-derived physical inactivity. In this context, a vicious circle is often identified in which pain in the lumbopelvic area [17,49] leads to physical inactivity and thus to muscle deconditioning that potentially affects deep abdominal muscles (with a postural function), which, in turn, might jeopardize motor control of the lumbopelvic area [44], increasing pain and decreasing the capacity to carry out their occupational roles [21]. In particular, recent findings of our research group have identified a number of physical impairments in women with endometriosis, including reduced pressure pain thresholds (PTTs), flexibility, body balance, muscle thickness and/or muscle strength of the lumbopelvic and abdominal regions; lower lumbopelvic stability and functional capacity; and increased fatigue when compared with healthy women [18,44]. In spite of the fact that few studies have recently reported benefits of exercise on HRQoL in endometriosis patients [40,50], none of them have been focused on the stabilization of the lumbopelvic area. Thus, the global aim of the ‘Physio-EndEA’ study will be to explore the short- and long-term effects of a supervised tailored therapeutic exercise program on the HRQoL and health-related fitness on women with a clinical diagnosis of endometriosis and a history of clinical symptoms. This therapeutic exercise program has been designed to improve tolerance to exertion, endurance and flexibility, paying special attention to the lumbopelvic area in order to mitigate pain associated to a poor motor control. The present methodological article describes the study design, procedures and methods that will be conducted in this project.

## 2. Materials and Methods

### 2.1. Study Design and Setting

**Figure 1:**
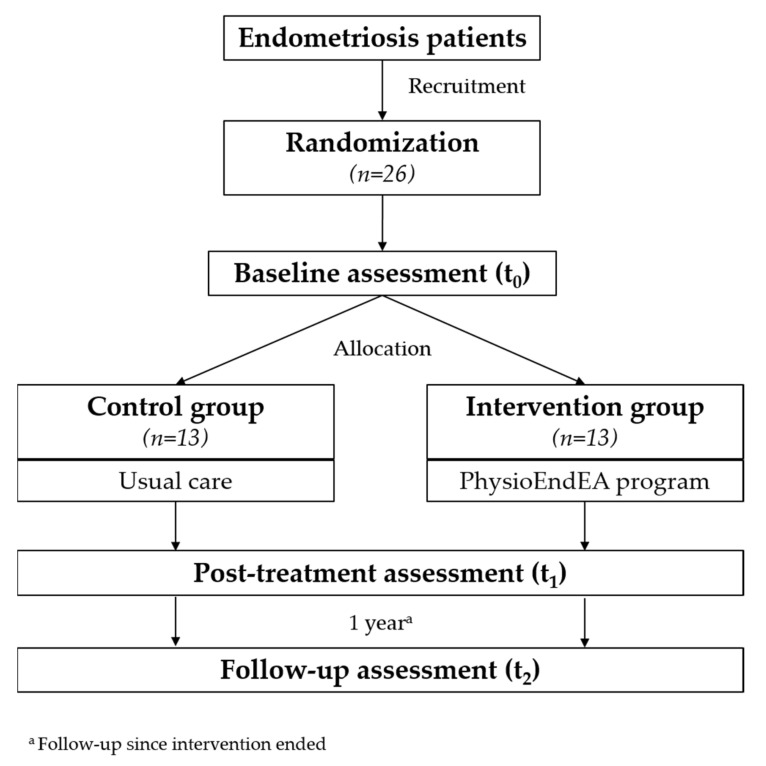
Flow diagram of participant recruitment during the trial according to SPIRIT.

Encompassed in a multidisciplinary research project (EndEA, Endometriosis y Exposición Ambiental), the ‘Physio-EndEA’ study is a parallel-group randomized non-inferiority trial (ClinicalTrials.gov, NCT03979183) based on a 1 + 8-week exercise program that will be conducted in 26 patients diagnosed with endometriosis (*n* = 13 experimental group vs. *n* = 13 control group) in Granada (Spain). The organizational and participants flow is presented in Figure 1. Primary and secondary outcomes will be assessed at baseline and immediately after intervention. Moreover, we will also evaluate the long-term impact of this exercise program by a final assessment 1 year after intervention with similar characteristics to the post-program evaluation. Physio-EndEA will be carried out at CUIDATE unit (http://csaludable.ugr.es/pages/dossierultimo/%21, accessed on 15 June 2021), a research unit for rehabilitation in oncology and related diseases. The protocol has followed the recommendations of the Standard Protocol Items: Recommendations for Intervention Trials (SPIRIT) checklist [51,52], and Figure 1 shows the SPIRIT diagram. This study was approved by the Clinical Research Ethics Committee of Granada, Government of Andalusia, Spain (code: 0792-N-18). ‘Physio-EndEA’ study was registered with ClinicalTrials.gov (code: NCT03979183).

### 2.2. Eligibility Criteria

The inclusion criteria for participants allocated to both control and intervention groups are as follows: To become a participant in this study, women need to declare a premenopausal status, hold a clinical diagnosis of endometriosis (by laparoscopy or magnetic resonance imaging), have a clinical history of endometriosis-related symptoms, be able to walk without assistance and to read and write enough, be capable and willing to provide consent and to be interested in improving their lifestyle.

However, we will exclude those women with acute or terminal illness, a recent fracture in any upper or lower extremity (<3 months), disc herniation and any chronic disease or orthopaedic issues that would interfere with her ability to participate in a physical activity program, as well as those that express unwillingness to complete the study requirements and those involved in other exercise program.

### 2.3. Intervention

#### 2.3.1. Exercise Intervention Group

‘Physio-EndEA’ is a 9 (1 + 8)-week supervised tailored program of therapeutic exercise that incorporates global and lumbopelvic exercises that was designed by a multidisciplinary team. It combines aerobic, resistance and stretching exercises with a core stabilization perspective. The intervention will be performed in small groups of 4–6 participants, training 90 min/session twice a week. Sessions are designed, carefully supervised, guided and instructed by a qualified group of physiotherapists and physical exercise specialists with more than 5 years of experience in therapeutic exercise in oncology and related diseases.

The intervention planning is shown in Table 1. The first part of the program, with a single week duration, consists of individual sessions for initial training of motor control by using both rehabilitative ultrasound imaging (RUSI) [53] and a Stabilizer^®^ Pressure Biofeedback (Chattanooga, Hannover, Germany). For that, patients will be initially RUSI-guided to selective pre- or co-activate both TrA and pelvic floor muscles through the abdominal drawing-in manoeuvre (ADIM), as previously suggested [54,55]. This method facilitates both patient learning and clinician verification of TrA muscle recruitment pattern. After that, a trained physiotherapist teaches a sort of stabilization exercises with and without Stabilizer^®^. Patients are invited to repeat this list of exercises twice daily (>5 min each) during that week. Patient will not progress to the second part of the program until the physiotherapist verifies motor control with RUSI through the ability to pre-/co-activate and maintain contraction of TrA and pelvic floor muscles during basic lower-limb exercises. Moreover, during the following 2 weeks, patients are encouraged to continue with home-based biofeedback exercises.

The second part of the intervention will lasts 8 weeks with group sessions twice per week focused on health-related fitness and motor control improvement. Each session will be divided into three sections: (1) an initial period with warm-up exercises (10 min); (2) a main period with aerobic (20–40 min) (continuous brisk walks), stretching (10–15 min) and lumbopelvic stabilization exercises (30–35 min) (taking into consideration the instructions previously learnt about motor control); and a final cool-down period of breathing and relaxation exercises. Details of the specific lumbopelvic stabilization exercises that these participants will perform are summarized in Table 1’s footnotes. Increases in both duration and intensity for aerobic exercises will be carried out based on fatigue perception. Alternatively, duration, intensity and number of repetitions for resistance exercises will be sequentially increased over time based on the adequate motor control of the lumbopelvic area, as well as fatigue perception. For that, by using the Borg Fatigue Scale [56], the physiotherapist (aided by two technicians) will daily inform about the maximum level of perceived fatigue that each patient must assume (from <11 at initial week to 13–14 at the end of the program), and thus, the women will increase or decrease the exercise intensity accordingly. For that purpose, women will be previously instructed with this scale to appropriately report their current perceived fatigue.

Adherence is defined based on the patient’s attendance to the sessions, considering as dropouts when they do not participate in at least 12 (75%) of 16 scheduled sessions. To maximize adherence, some strategies will be implemented, including adapted timetables to patient needs, music in all sessions and telephone calls following missed sessions.

#### 2.3.2. Usual Care/Control Group

Participants randomly assigned to the control group (*n* = 13) will receive the usual treatment, which is stipulated by their gynaecologist. Additionally, during the initial evaluation session, the trained physiotherapist assessor will offer general advice about the positive effects of physical activity and a healthy lifestyle for their HRQoL. For ethical reasons, control participants will be given the opportunity to participate in an exercise program with the same characteristics of this therapeutic exercise intervention after completion of this study, although their results will not be included in the analyses.

### 2.4. Outcome Assessment

Baseline, immediately after intervention and long-term evaluation, will be carried out in the CUIDATE unit, a platform for physiotherapy research in oncology and related diseases. Sessions will be conducted by a trained technician from the research group with 5 years of experience in taking these measurements and who will be blinded to patient group. In order to minimize the influence of menstrual cycle variability on study results, outcome assessment will be performed between days 2 and 10 of the menstrual cycle in women who are not using hormonal contraceptives. Table 2 summarizes primary and secondary outcomes that will be assessed, together with the list of validated instruments that will be used during the assessment.

#### 2.4.1. Primary Outcome: Health-Related Quality of Life

HRQoL will be addressed through the Spanish version of the Endometriosis Health Profile-30 (EHP-30) [57,58], a 30-item, disease-specific tool to evaluate HRQoL in women with endometriosis with high reliability (Cronbach’s α ranged between 0.79 and 0.97). It contains a total of five domains (pain, control and powerlessness, emotional well-being, social support and self-image), and items within each domain, answered on a 5-point Likert-type scale, are summed and transformed to a percentage scale. Scoring ranges from 0 to 100, with higher scoring representing a worse health status.

#### 2.4.2. Secondary Outcomes

1. Pain

Algometry will be used to measure PPT [59] levels in abdomen, pelvis and lower back regions based on previously published protocols for the evaluation of this area [17,49,60] through an electronic algometer (Somedic AB, Farsta, Sweden). For this purpose, an approximate rate of 30 kPa/s will be applied with a 1 cm^2^ probe. PPTs will be bilaterally assessed. Seven points, focused on abdominal and pelvic pain [49,60], and two additional points from the lower back region [17,60], will be tested. PPTs in the abdominal wall will be assessed in the following locations: the supraumbilical point is bilaterally assessed 3 cm above the umbilical point inside the hemiclavicular line (the lateral border of each rectus muscle); the infraumbilical point is bilaterally assessed 3 cm below the umbilical point inside the hemiclavicular line. The pelvic region will be assessed using 3 points: just above the pubis and both inguinal ligaments at its midpoint in the hemiclavicular line. The lower back area will be also assessed bilaterally, at the level of the fifth lumbar vertebrae (verified by ultrasound imaging). The algometer will be placed in the paraspinal area, in the middle of halfway on the belly of the erector spinae muscle (i.e., approximately 3 cm to the right or left of the marked spinae). Additionally, the second metacarpals of both sides will be assessed as distant points to the affected area. Prior PPT assessment, assessor will ask for participants to press the switch when they first feel a change from pressure to pain. The mean of three tests (intra-examiner reliability), spaced by 30 s, will be used for the analysis. A high reliability of pressure algometry has been found (interclass correlation coefficient 0.82–0.97) [61].

In addition to algometry, participants will be asked to indicate their current levels of chronic pelvic pain, dysmenorrhea, dyspareunia, dyschezia and dysuria through a Numeric Rating Scale (NRS). It is a 11-likert scale used for subjective pain estimation. It ranges from 0 (“no pain”) to 10 (“worst imaginable pain”). Participants are asked to select the whole number that best reflects the intensity of the pain that they feel at the moment of basal and final evaluation sessions. The NRS has shown to be a reliable and valid instrument to assess pain in endometriosis patients (Pearson product moment correlation = 0.96) [62,63].

The Pain Catastrophyzing Scale (PCS) [64] will be also used to assess catastrophic thinking related to pain. This 13-item scale contains 3 subscales: helplessness, rumination and magnification. All items are rated on a 5-point scale (0–4), and therefore, scores range from 0 to 52. The PCS has been shown to have adequate to excellent internal consistency (Cronbach’s α ranged from 0.87 to 0.93) [64].

2. Muscle thickness

Images of the abdominal wall and lumbar multifidus will be obtained using an ultrasound device (Samsung HM70A echograph, Samsung LA3-16AD Linear probe) with a 12 MHz linear probe and a depth of 5 cm. The thicknesses of the external and internal oblique (OE and OI, respectively), the transversus abdominal muscles (TrA) and the lumbar multifidus, as well as the width of the lumbar multifidus, will be assessed according to a previously described methodology [60]. Patients are placed in supine position with arms lined up with the trunk, and images are gathered when patients are relaxed and at the end of the expiration movement. Three measurements of both right and left muscles diameters will be recorded with a 2-min interval between trials. Probe will be set 2.5 cm anteromedial to the mid-point between the iliac crest and the costal margin on the mid-axillary line, where the fascial boundaries between TrA, OI and OE and the superior edge of the TrA fascia lie parallel [65]. The average of three trials will be used. Ultrasound imaging has been reported to be reliable for testing muscle thickness of TrA, OI and OE (interclass correlation coefficients > 0.85, 0.65 and 0.80, respectively) [66,67].

Lumbar multifidus assessment will be performed at the fifth lumbar vertebra, marking its spinal process. For that, lordosis of prone-positioned participants will be corrected using pillows below the abdominal area. Depth of lumbar multifidus will result from the greatest perpendicular anteroposterior distance from the processes transversus to posterior layer of the lumbar fascia. Width of lumbar multifidus will be recorded as the greatest horizontal distance between the lateral aspects of the spinous process and the fascial boundary of the longissimus muscle [60]. High reliability for ultrasound evaluation of lumbar multifidus has been reported (interclass correlation coefficient = 0.88) [67].

3. Strength of the lumbopelvic region

Endurance strength of abdominal muscles will be assessed by the muscle trunk flexor endurance test that evaluates the isometric endurance of trunk flexors. For that, women are placed in supine position with hips and knees flexed at 90 degrees, feet flat approximately 30 inches from the buttocks and arms extended with hands on knees without actually touching. Patients will be instructed to separate the trunk from the stretcher to the inferior angle of the scapula and maintain this position as long as possible. Time (in seconds) will be measured [68]. Higher scores represent better performance. It has been reported that this test has high reliability (interclass correlation coefficient (ICC) > 0.95) [69].

Isometric endurance of trunk extensors will be evaluated with the muscle trunk extensor endurance test that assess the isometric endurance of back extensor muscles. Patients lying in prone position with the lower extremities stand on the bed and fixed with a strap and the trunk and upper extremities hanging on in a horizontal position with arms folded and hand in touch with the contralateral shoulder. The bed border will coincide with the anterior superior iliac spines. Women will be asked to maintain this position as long as possible. Time (in seconds) will be measured, with higher scores reflecting better performance [70]. This test has high reliability in either symptomatic and non-symptomatic low back pain individuals (ICC > 0.77) [71].

4. Lumbar spine flexibility

Flexibility of the lumbar spine will be addressed with the original Schöber test [72]. For that purpose, the physiotherapist will localize the lumbosacral junction and mark it. A second mark will be drawn 10 cm above the first one when the patients are in erect position. This distance will be measured in flexion position, with higher differences between erect and flexion position representing better flexibility.

5. Body balance

Flamingo test will be used to assess body balance. Standing on a beam with shoes removed, participants are asked to balance on the preferred leg while the free leg is flexed at the knee and the foot of this leg is close to the buttocks. Test is repeated with the contralateral leg. The number of trials needed to complete 30 s of the static position is recorded. The average of both legs was used in the analysis [73]. Lower scores reflect better balance.

6. Functional capacity

The 6-Minute Walking Test (6MWT), which showed good reliability (ICC = 0.97) [74], will be used to determine the maximum distance (in metres) that each patient can walk in 6 min. Participants will be previously familiarized with the test protocol, ‘walking as far as they can in 6 min’, increasing and decreasing the speed voluntarily. Standardized phrases of encouragement will be given by the examiner during the 6MWT. Walking pace, defined as at least one foot being weight-bearing at all times, will be monitored during the entire task. Blood pressure, heart rate, oxygen saturation and Borg Fatigue Scale will be assessed before and after the 6MWT. Higher scores represent better performance.

7. Body composition

Anthropometric measurements (including height, weight and body mass index) and body composition (skeletal muscle mass and percentage of body fat) will be recorded. An impedance meter (InBody 720; Biospace, Seoul, South Korea) will be used for these measurements, which has exhibited high reliability in previous studies [75]. Patients will be instructed to avoid eating and drinking during the hour prior to measurement. Moreover, they will be also invited to take off any wristwatch, wristband, necklace or belt during the measurement. Finally, the hour of the day of the measurement will be recorded.

8. Fatigue

Endometriosis-related fatigue will be evaluated with the Spanish version of the Piper Fatigue Scale-Revised. Although it was originally developed to assess cancer-related fatigue [76], it has been also used to assess fatigue in other musculoskeletal disorders such as heart failure [77] or gynaecological problems [78,79], including endometriosis [18,20]. This 22-item tool is grouped in 4 dimensions (behavioural/severity, affective meaning, sensory and cognitive/mood) and scores ranges from 0 to 10, with lower scores reflecting a better performance. PFS-R has high reliability (Cronbach’s α = 0.96) [76].

9. Quality of sexual life

To assess sexual function, an important aspect of the quality of life of a person, we will use the validated Female Sexual Function Index (FSFI) questionnaire [80]. It is a self-reported 19-item questionnaire that covers six dimensions, including desire, arousal, lubrication, orgasm, satisfaction and pain. Scores of each domain are calculated as the sum of the individual items contained in each domain and multiplied by the domain factor. Finally, all domain scores are summoned to calculate the overall score, ranging from 2 to 36, with higher values representing a better sexual function, considering that patients with a FSFI total score below 26 are sexually dysfunctional, whereas those scoring at or above this cut-off score are categorized as sexually functional [81]. This questionnaire has good reliability (Cronbach’s α > 0.82 for all domains) [81].

10. Gastrointestinal quality of life

The Gastrointestinal Quality of Life Index (GQLI) is a validated, 36-item, self-administered questionnaire that addresses 5 domains (digestive symptoms, physical status, emotions, social dysfunction and effects of medical treatment) [82]. Scores of individual items (0 being the worst appreciation and 4 the best appreciation) are summed in order to calculate the global score. It ranges from 0 to 144, with higher scores indicating a better gastrointestinal quality of life. It is considered that patients with a total score below 100 suffer from gastro-intestinal diseases, while healthy controls usually obtain punctuations above 126 [83]. This index has shown good reliability (Chronbach’s α > 0.90) [82] and has been previously applied to endometriosis patients [22].

11. Sleep quality

The validated Pittsburgh Sleep Quality Index (PSQI), previously used in endometriosis patients [14], will be used to address the quality and patterns of sleep of the women [84]. This 19-item instrument has moderate-high reliability (Cronbach’s α > 0.80) [85]. It is composed of seven “components” (subjective sleep quality, sleep latency, sleep duration, habitual sleep efficiency, sleep disturbances, use of sleeping medication and daytime dysfunction), each of them ranging from 0 to 3. The sum of component’s scores leads to the global score and thus ranges from 0 to 21, with higher scores indicating poor sleep quality. It has been proposed that a total score ≤ 5 indicates good sleep quality while a total score > 5 indicates poor sleep quality [84].

### 2.5. Recruiting and Participant Timeline

Gynaecologists from both University hospitals (San Cecilio and Virgen de las Nieves, Granada) will provide eligible patients. Women interested in the study will receive more information about the study objectives, evaluation protocol and procedures and will sign informed consent before undertaking baseline assessment. Potential participants will need to provide a copy of laparoscopic or magnetic resonance imaging findings confirming a diagnosis of endometriosis.

### 2.6. Sample Size

The estimated sample size was determined for the primary outcome variable (HRQoL-pain) using the EHP-30 questionnaire and calculations from a previous study to detect a mean difference between control and intervention groups of 24.7 (28.4 ± 18.7 vs. 3.66 ± 18.4; Gonçalves, Barros and Bahamondes [40]). Assuming an α error of 0.05, a power of 85% and an effect size of 1.34 (based on the results of the reference study), we need a sample of 10 participants for each group. Assuming a possible 30% dropout rate, we will recruit a minimum of 13 participants per group (*n* = 26). G*Power v. 3.1 (Düsseldorf University, Düsseldorf, Germany) was used to calculate the sample size.

### 2.7. Randomization and Blinding

Once the baseline evaluation will be completed, participants will be included in either the therapeutic exercise intervention or the control group (1:1). For that, a computer-generated sample randomization sequence will be created by a researcher not involved in the clinical part of this study (Epidat 3.4, Xunta de Galicia, Spain). Once known, the primary investigator will communicate the assigned group to each participant. Therefore, although practitioners and participants will not be blinded, other researchers (assessors, statisticians and data managers) will be blind to group allocation. Participants will be asked not to mention any details of their treatment or their group allocation to the assessor(s) (responsible for evaluation sessions). If blinding is compromised, another assessor will be contacted and will complete the data collection.

### 2.8. Data Collection

As shown in Figure 1, women will be evaluated in three moments: baseline assessment (t0), immediately after program (t1) and one year after completion of treatment (t2). Assessment will take approximately 90 min. Information gathered will be kept under lock and key. To maintain the confidentiality of personal information, a coded ID number will be assigned to each participant.

### 2.9. Data Analysis

Since this study aims to determine the potential benefits of therapeutic exercise on the HRQoL of endometriosis patients, statistical analyses will be performed on an intention-to-treat basis. Only those participants that would have attended at least 75% of the sessions and completed at least two assessments (baseline and one evaluation of the two follow-ups) will be included in the analyses. The worst-case value will be used to replace missing data, after a previous reported procedure.

Mean and standard deviation will be used to describe variable scores at baseline and both follow-up measurements (postprogram and 1 year). Additionally, the Shapiro–Wilk test will be used to assess the normality of the distribution of the variables. To evaluate the ability of the randomization process to avoid differences between groups at baseline, Student’s t-test and chi-square test will be used to compare continuous and categorical data, respectively. An equivalent statistical approach will be conducted in case of non-parametrical data (Mann–Whitney and Kruskal–Wallis tests).

To evaluate the influence of treatment on outcome scores, a repeated-measure ANCOVA between the three time points (baseline, postprogram and 24 weeks later) will be used to examine the between-group and within-subject differences. Patient age, years since diagnosis, education level, marital status and the presence of heavy bleeding during the menstruation period will be used as covariates. To complete the analysis, we will report effect size (Cohen d) and level significance attending to interaction effects (group x time) to examine intergroup effects and to determine whether these were negligible (d < 0.2), small (0.2 < d > 0.5), moderate (0.5 < d > 0.8) or large (d > 0.8).

The statistical significance will be set at α = 0.05. Statistical analyses will be conducted on the Statistical Program for Social Sciences (IBM, SPSS 24.0).

## 3. Discussion

This paper describes the protocol developed by a multidisciplinary research team of experts in physical therapy and gynaecology that aims to determine the potential benefits of a 1 + 8-week supervised program of supervised tailored therapeutic exercise oriented toward core stabilization on the HRQoL of women diagnosed with endometriosis and a history of clinical symptoms, given the preliminary positive impact observed for this physiotherapeutic approach in patients with similar affected area [41,86,87].

Although the burden of endometriosis symptoms is highly variable between patients [16], it is well known that almost all women with endometriosis suffer from chronic pelvic pain, which may cause variable limitations in their daily lives [31]. Therefore, in cases of a severe burden of symptoms, they may lead to a disability status. As mentioned above, usual medical care, generally consisting of analgesics and oral contraceptives in combination (or not) with surgical interventions, is clearly insufficient for a satisfactory pain control in the majority of endometriosis patients. In this regard, physical therapy, highly specialized in chronic pain management, might offer potential benefits that, in combination with usual medical care, may lead to a better pain control. Moreover, this therapeutic approach might also imply a meaningful reduction of direct and/or indirect costs of endometriosis that now reach up to >USD 12.000 and >USD 15.000 per patient per year in the USA, respectively [35].

Until now, similar approaches have revealed significant benefits on the HRQoL for patients with a lumbopelvic affectation such as those suffering chronic low-back pain [88] or colorectal cancer survivors [41,86]. Concerning endometriosis patients, only few studies have revealed significant improvements in endometriosis-related HRQoL and/or pain control after physical therapy treatments such as neuromuscular electrical stimulation [38], pulsed high-intensity laser therapy [39], visceral osteopathy [37] or yoga [40]. However, this is, to our knowledge, the first randomized controlled trial specifically designed to explore the contribution of a supervised tailored program of therapeutic exercise with special emphasis on the lumbopelvic area on physical and occupational impairments, as well as on the HRQoL of women with endometriosis and a clinical history of symptoms. In this regard, it is acknowledged that, apart from a neuromuscular control of lumbopelvic stability (i.e., a sensory input that alerts the central nervous system about interaction between the body and the environment, providing constant feedback and allowing refinement of movement), deep abdominal muscles (specifically TrA) and lumbar multifidus, whose thicknesses and strengths were found to be reduced in women with endometriosis [18,44], are the main muscles responsible for lumbopelvic stability [89]. Moreover, it has been reported that TrA and the lumbar multifidus are recruited prior shoulder and leg movements in healthy people (30 and 110 ms, respectively) [90,91]. Hence, the co-contraction of deep trunk muscles connects the stability of the upper and lower body via the abdominal portion, and thus, there is a physiologic temporal sequence of core muscle recruitment prior to activation of the muscles of the extremities during many daily activities, allowing a more stable base for muscle activation and improving the firmness of the lumbar spine. This anticipated pattern provides a “proximal stability for distal mobility”, which is of particular importance for symptomatic women with endometriosis during the daily life activities such as household, work and leisure demands [89].

## 4. Conclusions

The establishment of this type of intervention could benefit the HRQoL of symptomatic women with endometriosis. Moreover, it might reduce the direct and indirect costs of this health problem. Consequently, findings derived from the ‘Physio-EndEA’ study will help the Health Systems to design cost-effective strategies for health promotion among a numerous group of affected women, whose prevalence is estimated in 1 to 10 women in childbearing age, i.e., approximately 176 women worldwide [2,3].

## Figures and Tables

**Table 1:** Supervised ‘Physio-EndEA’ exercise intervention program.

	Exercise	Week 1	Week 2	Week 3	Week 4	Week 5	Week 6	Week 7	Week 8	Week 9
Part 1	US-guided training of core stabilization	√								
	Biofeedback exercises	√								
	Home-based biofeedback exercises	√	√	√						
Part 2	Warm-up		10 min	10 min	10 min	10 min	10 min	10 min	10 min	10 min
	Lumbopelvic stabilization exercises ^1^		1 set of 8–10 reps	1 set of 12 reps	2 sets of 8 reps	2 sets of 10 reps	1 set of 12 reps	2 sets of 12 reps	3 sets of 8 reps	2 sets of 10 reps
	Aerobic exercise		20 min	20 min	20 min	30 min	35 min	40 min	40 min	40 min
	Stretching exercises		10 min	10 min	10 min	10 min	10 min	10 min	10 min	10 min
	Cool-down ^2^		10 min	10 min	10 min	10 min	10 min	10 min	10 min	10 min

^1^ Lumbopelvic stabilization exercises includes: Roll up-Roll down, one leg circle, side kicks, saw, hundred and leg pull front. ^2^ Breathing and relaxation exercises.

**Table 2:** ‘Physio-EndEA’ evaluation protocol scheme.

Assessment	Instrument ^1^	Baseline	Post-Intervention	Detraining
Informed consent	-	√		
Sociodemographic/clinical data	Ad-hoc questionnaire	√		
Body composition	Inelastic tape	√	√	√
	Impedance meter	√	√	√
Health-related quality of life	EHP-30	√	√	√
Pain	Algometry	√	√	√
	NRS	√	√	√
	PCS	√	√	√
Muscle thickness	Ultrasound imaging	√	√	√
Muscle strength	Muscle trunk flexor endurance test	√	√	√
	Muscle trunk extensor endurance test	√	√	√
	Handgrip dynamometry	√	√	√
	Back strength dynamometry	√	√	√
Flexibility	Schöber test	√	√	√
Body balance	Flamingo test	√	√	√
Functional capacity	6MWT	√	√	√
Fatigue	PFS-R	√	√	√
	Borg Scale	√	√	√
Anxiety and depression	HADS	√	√	√
Quality of sexual life	FSFI	√	√	√
Gastrointestinal quality of life	GQLI	√	√	√
Sleep quality	PSQI	√	√	√

^1^ EHP-30: Endometriosis Health Profile-30; NRS: Numeric Rating Scale; PCS: Pain Catastrophyzing Scale; 6MWT: 6-min walking test; PFS-R: Piper Fatigue Scale-Revised; HADS: Hospital Anxiety and Depression Scale; FSFI: Female Sexual Function Index; GQLI: Gastrointestinal Quality of Life Index; PSQI: Pittsburgh Sleep Quality Index.

## Data Availability

Members of the research group will encourage the publication of future results of the study in scientific papers in an open access journal and will try to do so if possible. Furthermore, access to the full protocol and data will also be encouraged.
